# Survival After Orbital Exenteration for Primary Cutaneous Squamous Cell Carcinoma: A Retrospective Cohort Study

**DOI:** 10.1245/s10434-024-16854-w

**Published:** 2025-01-13

**Authors:** Alexander Murray-Douglass, Lachlan Crawford, Justin Hunt, Darryl Dunn, Brett G. M. Hughes, Charles Lin, Carly Fox

**Affiliations:** 1 Department of Plastic and Reconstructive Surgery, Royal Brisbane and Women’s Hospital, Brisbane, Queensland Australia; 2 Faculty of Medicine, The University of Queensland, Brisbane, Queensland Australia; 3 Department of Medical Oncology, Royal Brisbane and Women’s Hospital, Brisbane, Queensland Australia; 4 Department of Radiation Oncology, Royal Brisbane and Women’s Hospital, Brisbane, Queensland Australia

**Keywords:** Skin neoplasms, Cutaneous squamous cell carcinoma, Orbit evisceration, Orbital exenteration, Immunotherapy

## Abstract

**Background:**

Locally advanced periorbital cutaneous squamous cell carcinoma (cSCC) may require orbital exenteration, which is highly morbid. As immunotherapy develops, orbit preservation may become widespread, and data benchmarking survival with current standard-of-care surgery and radiotherapy are essential to the integration of this emerging method into modern treatment paradigms. This study aimed to determine the survival of patients after orbital exenteration for cSCC and investigate contributing factors. It was hypothesized that postoperative radiotherapy would be associated with improved survival.

**Methods:**

This was a retrospective cohort study of patients with T3 and T4 cSCC undergoing orbital exenteration. Survival analysis was performed using Cox proportional hazards.

**Results:**

The study enrolled 40 patients with a median age of 61.5 years who met the criteria. None of the patients had received preoperative radiotherapy. Age (hazard ratio [HR], 1.09; *p* = 0.019) and residual disease (HR, 9.00; *p* = 0.003) were associated with worse survival. Postoperative radiotherapy (HR, 0.003; *p* < 0.001) was associated with improved survival. Perineural, lymphovascular, and bony invasion and T and N stage were not associated with survival. Survival with postoperative radiotherapy was 94 % at 1 year, 87 % at 2 years, and 84 % at 5 years.

**Conclusions:**

The oncologic outcomes of orbital exenteration with postoperative radiotherapy for locally advanced head and neck cSCC are good. However, amelioration of the morbidity caused by resection of the eye would be ideal. Data to support immunotherapy as a sole therapy are currently limited, but a combination of neoadjuvant immunotherapy and surgical treatment may facilitate orbit-preserving treatment in the future.

Keratinocyte cancers, including cutaneous squamous cell carcinoma (cSCC), are the most common cancers in the world. Estimating cSCC prevalence is difficult due to its lack of inclusion in cancer registries and high prevalence, but approximately 1 in 10 Americans^1^ and 1 in 5 Australians^2^ can expect to have cSCC in their lifetime.

Most cSCCs occur in the head and neck^3^ due to long-term exposure to ultraviolet radiation from the sun.^4^ In Australia, age-standardized mortality rates for keratinocyte cancer are stable, at about 3 per 100,000 person-years.^5^ Also, cSCC may be costly, with an average hospital duration of 5.8 days and a cost of USD$66,841.^6^

Early cSCC usually can be cured by simple surgical excision,^7^ but more advanced disease may require more significant surgery with or without postoperative radiotherapy. Locally advanced cSCC invading into the orbit is an indication for orbital exenteration, a procedure in which the contents of the orbit are surgically removed.^8^ Orbital exenteration is a morbid procedure, with significant functional and aesthetic consequences including loss of vision, facial disfigurement, functional impairment of the remaining eye, psychological distress, and general surgical complications.^9^ Postoperative radiotherapy also is commonly indicated for the same patient group that requires orbital exenteration.^10^

Immunotherapy has revolutionized oncology by weaponizing the body’s own defense, the immune system, enabling it to recognize and attack cancer cells more effectively. For cSCC, immune checkpoint inhibitors have shown great promise in two major clinical trials of locally advanced disease. The EMPOWER-CSCC-1 phase 2 clinical trial^11^ showed the efficacy of cemiplimab, an anti-programmed cell death protein 1 (PD-1) monoclonal antibody, against locally advanced cSCC. Of 78 patients, 34 (44 %) experienced confirmed response during a median follow-up period of 9.3 months, with 23 (68 %) of the responses remaining at 6 months. Subsequently, the KEYNOTE-629 phase 2 clinical trial^12^ showed the efficacy of pembrolizumab, another anti-PD-1 monoclonal antibody, against advanced cSCC. Among the locally advanced disease cohort, a confirmed response was seen in 27 patients (50 %) during a median follow-up period of 14.9 months, with 84.1 % of the responses estimated to last 12 months or longer.

More recently, immunotherapy in the neoadjuvant space has been vindicated, with preliminary results from a phase 2 trial involving up to four cycles of neoadjuvant cemiplimab before planned resection for predominantly head and neck cSCC.^13^ This trial showed a pathologic complete response in 40 patients (51 %). Notably, two patients avoided planned orbital exenteration after neoadjuvant immunotherapy.

Also, more recently, the initial results of the De-Squamate trial for resectable cSCC have been presented.^14^ In this trial, 27 patients received at least two cycles of neoadjuvant pembrolizumab, with 17 patients (63 %) having de-escalation of planned surgery or postoperative radiotherapy. These trials indicated that immunotherapy may provide an eye-sparing alternative to orbital exenteration for locally advanced cSCC in the head and neck region.

The current study aimed to provide an updated assessment of traditional management of locally advanced head and neck cSCC with orbital exenteration and postoperative radiotherapy to serve as a comparison for future studies of immune agents. It also aimed to determine the survival of patients after orbital exenteration for T3 and T4 primary cSCC and to investigate factors that might contribute to survival. It was hypothesized that age, tumor (T) and nodal (N) stage, and the presence of perineural, lymphovascular and bony invasion as well as the presence of residual disease after surgery would predict mortality and that postoperative radiotherapy would be associated with improved survival.

## Materials and Methods

### Design

This study was exempted from ethics review by the Royal Brisbane and Women’s Hospital (RBWH) Human Research Ethics Committee (LNR/2020/QRBW/63089). The study used a retrospective cohort study design in accordance with the Strengthening the Reporting of Observational Studies in Epidemiology (STROBE) statement.^15^

### Setting and Participants

The study analyzed data collected at the RBWH in Brisbane, Australia between May 2009, and February 2020. Follow-up data were collected until December 2023. Patients were identified by a search of the RBWH Operating Room Management Information System (ORMIS) database. This database was searched using International Classification of Diseases, 10th revision (ICD-10) codes to identify all patients who underwent orbital exenteration at the RBWH. The study enrolled patients if they underwent orbital exenteration as characterized by ICD-10 codes 42536-00 through 42536-05, inclusive. The medical records of these cases then were searched to identify whether they met the subsequent inclusion criteria.

Staging of non-melanoma skin cancer (NMSC) is described by the American Joint Committee on Cancer (AJCC) staging system and incorporates tumor stage (T). In the seventh edition, T3 is a tumor of the head and neck with invasion of the maxilla, mandible, orbit, or temporal bone, and T4 is a tumor with invasion of the skeleton (axial or appendicular) or perineural invasion of the skull base.^16^ In 2018, the eighth edition of the AJCC staging system^17^ was introduced into clinical practice. However for the purpose of this retrospective study, we used the seventh edition because it was in use throughout the majority of our study period.

To be included in the study, patients must have had exenteration to treat a T3 or T4^16^ primary cutaneous SCC with orbital reconstruction in the same operation using any reconstructive method. The study excluded patients if orbital exenteration was for treatment of any other orbital malignancy or other indication or if they received orbital enucleation or evisceration without exenteration.

### Data

Once patients were selected, their medical records were searched to identify their demographic details including age and sex, cancer details (stage), reconstruction details, follow-up details (mortality status, successive procedures, complications, and postoperative radiotherapy), and rehabilitation details (uptake of orbital rehabilitation with an orbital prosthesis). The primary outcome of interest was patient survival. The secondary outcomes included complications associated with treatment, reconstructive methods, and eventual orbital rehabilitation.

### Statistical Analysis

Survival analysis was performed using a Kaplan-Meier survival curve, with multivariate analysis using the Cox proportional hazards model. Age, T and N stage, residual disease, postoperative radiotherapy, and the presence of perineural, lymphovascular, or bony invasion were considered from the outset as independent variables and included in the multivariate analysis. Stata IC version 16.1 for Mac (StataCorp, College Station, TX, USA)^18^ was used for statistical analysis.

## Results

**Fig. 1:**
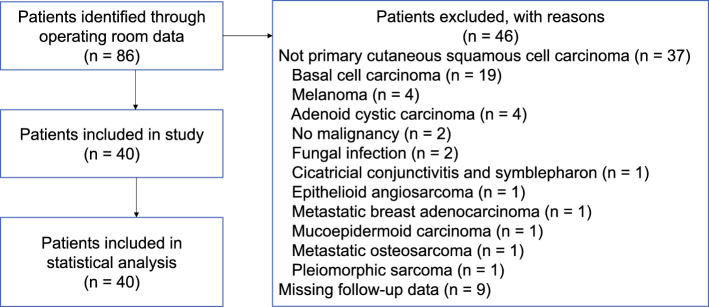
Patient selection and treatment flowchart

The study identified 86 patients as having undergone orbital exenteration at the RBWH between May 2009 and February 2020. A summary of the patient selection process with exclusions is shown in Fig. 1. After excluding 46 patients who did not meet the inclusion criteria, the study enrolled 40 patients. The demographic details of these patients are presented in Table 1. No patients had previously received radiation therapy. Histologic analysis showed perineural invasion (PNI) in 27 patients (68 %), lymphovascular invasion in four patients (10 %), and bony invasion in 22 patients (55 %). With regard to excision margins, 28 patients (70 %) had no residual disease at surgical margins (R0 resection), 3 patients (8 %) had microscopic residual disease (R1 resection), and 9 patients (23 %) had macroscopic residual disease (R2 resection), with 32 patients (80 %) going on to have postoperative radiotherapy.

**Table 1:** Patient demographics.

Characteristic	Value (*n* = 40) *n* (%)
Median age at time of surgery: years (IQR)	61.5 (51–75)
Sex	
Female	9 (23)
Male	31 (78)
ECOG status	
0	13 (33)
1	19 (48)
2	3 (8)
3	5 (13)
T stage	
T3	18 (45)
T4	22 (55)
N stage	
N0	33 (83)
N1	3 (8)
N2	4 (10)
N3	0 (0)
Bony resection	27 (68)
Free flap reconstruction	29 (73)

IQR, interquartile range; ECOG, Eastern Cooperative Oncology Group

**Fig. 2:**
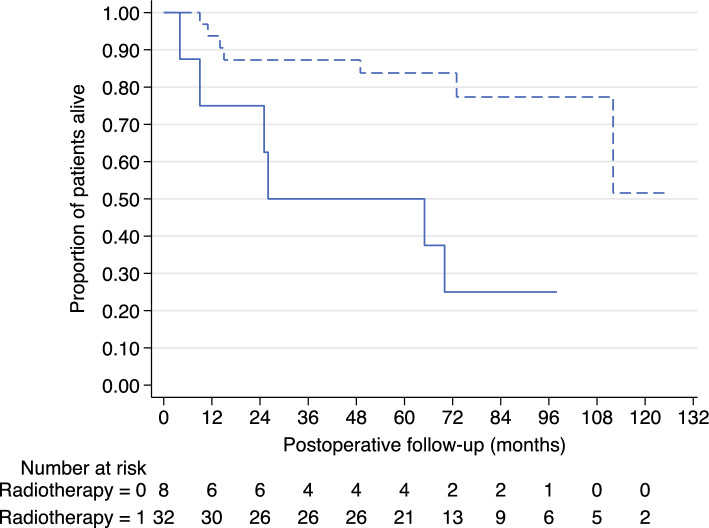
Kaplan-Meier survival curve showing proportion of patients alive at a given time point in months after orbital exenteration, stratified by the use of postoperative radiotherapy. Solid line is no radiotherapy (radiotherapy = 0). Dashed line is radiotherapy (radiotherapy = 1). The numbers of patients at risk at each time point are presented below the graph

The regression model statistically significantly predicted survival (*n* = 40, LR X^2^ (8) = 28.74; *p* < 0.001). Of the independent variables assessed, age (hazard ratio [HR], 1.09, standard error [SE], 0.04; *p* = 0.019) and residual disease (HR, 9.00; SE, 6.72; *p* = 0.003) were associated with worse survival. Postoperative radiotherapy (HR, 0.003; SE, 0.005; *p* < 0.001) was associated with improved survival. In our cohort, T stage, N stage, PNI, lymphovascular invasion, and bony invasion were not associated with survival (*p* > 0.05). For those who had not received postoperative radiotherapy, the survival rates were 75 % at 1 year, 75 % at 2 years, and 50 % at 5 years. For those who had received postoperative radiotherapy, the survival rates were 94 % at 1 year, 87 % at 2 years, and 84 % at 5 years (Fig. 2).<F2>

Follow-up evaluation was performed for a median of 65 months (interquartile range, 37–85.5 months). Complications of any kind were seen in 14 patients (35 %), with 9 patients (23 %) requiring surgical treatment of a complication. Partial or total flap failure was the most common complication in five patients (13 %), with infection in three patients (8 %) and hematoma in two patients (5 %). Recurrence of any kind occurred in nine patients (23 %), with seven cases (18 %) of local or regional recurrence and three distant metastases (8 %). Two patients (5 %) underwent re-excision for recurrent disease. One patient (3 %) received adjuvant immunotherapy. Orbital rehabilitation with a prosthesis was achieved for four patients (10 %).

## Discussion

In our study, age, residual disease, and lack of postoperative radiotherapy were associated with higher mortality after orbital exenteration for cSCC. The 5-year survival rates were 84 % for the patients who had postoperative radiotherapy and 50 % for the patients who did not. Keratinocyte cancer of the head and neck has previously demonstrated characteristics correlated with survival. Findings have shown the positive final surgical margin to be correlated with mortality,^19^ as in our study. However, previous series with much more heterogeneous samples failed to detect a difference in survival based on residual tumor.^20,21^ Previous series also have failed to demonstrate a difference in survival based on the presence of PNI and postoperative radiotherapy.^22,23^ However, PNI is a known determinant of poor outcomes.^24,25^

Although oncologic results are quite acceptable with a combination of orbital exenteration and postoperative radiotherapy, morbidity remains high. Several factors play into this morbidity and can be optimized in turn. First, there are direct complications related to exenteration and associated reconstruction surgery. In our cohort, this accounted for a complication in 14 patients (35 %). Second, there are complications arising from postoperative radiotherapy, such as radionecrosis.

Furthermore, disease recurrence and progression also can be a cause of morbidity and mortality, although this has a low rate with current standard of care. Morbidity also stems from both the functional and aesthetic complications from loss of an eye. Major losses to peripheral vision on the affected side and stereopsis take up to 12 months of adaptation before a person can return to adequate function.^26^ In elderly patients, who commonly present with locally advanced cSCC, this is a serious issue that can predispose to falls, which have high mortality and morbidity.^27^ In our cohort, one major complication occurred after a patient fell postoperatively, leading to flap failure, exposure of bone, and cerebral infection. The psychological impact of orbital exenteration often is overlooked but can include loss of self-esteem, fear of loss of the remaining eye, poor eye contact, and social interaction, and may lead to social withdrawal and isolation.^9,26^

Primary treatment of orbital cSCC with cemiplimab has recently been assessed in a retrospective study in Israel.^28^ In this study, 13 patients with a median age of 76 years who had orbital invasion of cSCC and either were not surgical candidates or wanted to avoid orbital exenteration were offered primary immunotherapy with cemiplimab. These patients had a response rate of nine (69 %) and a complete response in seven (54 %) after a median follow-up period of 15 months. Of 13 patients, 4 died within the follow-up period, giving an approximate survival of 69 % at 15 months. This is lower than the current survival rates from standard exenteration and radiotherapy, but should be considered in the context of a much newer therapy that obviates the need for removal of the globe and vision. Furthermore, the sample had broader inclusion criteria for Eastern Cooperative Oncology Group (ECOG) status than many trials, including ECOG 4 for four patients (31 %).

Our cohort also had varied ECOG status, with eight patients (20 %) categorized as ECOG 2 or 3, the usual exclusion criteria for clinical trials. Regarding cSCC more generally, the final analysis of the locally advanced group from the EMPOWER-CSCC-1 trial of cemiplimab showed an overall survival of 92 % at 1 year, 83 % at 2 years, and 60 % at 5 years for unresectable patients.^29^

The ultimate treatment method is likely to be a combination of best-practice treatments, including neoadjuvant immunotherapy to debulk the tumor and possibly facilitate globe-preserving surgery, with or without postoperative radiotherapy. An example of this approach was presented in a case report of a recurrent T3N1 cSCC in a 72-year-old man treated with neoadjuvant cemiplimab and followed by lateral orbitotomy with globe-sparing tumor-debulking and reconstruction of the orbital rim.^30^ This paradigm has culminated in a recently published phase 2 trial of neoadjuvant cemiplimab in up to four cycles before previously planned surgical resection.^13^ The 2-year survival data are due for presentation later this year, but the 1-year data are available.^31^ Of 79 patients who received neoadjuvant cemiplimab, 70 proceeded to surgery. Pathologic complete response was observed in 40 patients (57 %), major pathologic response in 10 patients (14 %), pathologic partial response in 7 patients (10 %), and no response in 13 patients (19 %). Only 17 patients (24 %) were recommended for postoperative radiotherapy after resection and histologic analysis in this trial, which is low considering that the patients all had stages 2 to 4 cSCC. The estimated median event-free survival was 89 % (95 % confidence interval [CI], 79–94 %) at 12 months. In the pilot study before this trial, 11 of 20 patients preoperatively thought to require postoperative radiotherapy were subsequently not recommended for radiotherapy.^32^

Recently, preliminary results of the Australian government-funded De-Squamate phase 2 trial of treatment de-escalation with neoadjuvant pembrolizumab have been presented.^14^ The patients received two to four cycles of neoadjuvant immunotherapy and were reassessed at this time to determine whether treatment de-escalation was clinically appropriate. Of the 27 patients, 13 (48 %) demonstrated a complete clinical response, defined as a complete metabolic response combined with negative-mapping biopsies after neoadjuvant therapy. These patients were de-escalated and did not receive surgery or radiotherapy. A further four patients (15 %) with either clinical or radiographic residual disease or positive mapping biopsies underwent surgical resection, showing a pathologic complete response, and avoided postoperative radiotherapy. At this writing, none of these patients have experienced recurrence within a minimum 6-month follow-up period. There were two investigator-assessed treatment-related complications of grade 3 or greater (7 %). Publication of the results from this trial may elaborate on possible orbit-preserving cases.

Although it is unclear at this time how combination surgery and postoperative radiotherapy compare with neoadjuvant immunotherapy and surgery in terms of survival, several studies are currently underway that may shed light on this question. However, from first principles, the only possible way to reduce morbidity caused by radical surgical resection is to use therapy that can reduce the cancer burden in the neoadjuvant space before surgery, thereby reducing the extent of surgery required.

It is important to consider complications when determining whether a treatment paradigm should include novel therapies. Complication data from the EMPOWER-CSCC-1 trial^11^ assessing cemiplimab for advanced cSCC in 78 patients were reported according to the National Cancer Institute Common Terminology Criteria for Adverse Events (v4.03). Analysis showed any treatment-emergent adverse event in 77 patients (99 %), with fatigue as the most common adverse event in 32 patients (41 %). Grades 3 and 4 adverse events, which represent serious to life-threatening complications even if not related to treatment, occurred in 34 patients (44 %), with hypertension in six patients (8 %) and pneumonia in four patients (5 %). Six patients (8 %) discontinued treatment due to adverse events. Two patients (3 %) died. Regarding attribution of adverse events, the study investigators considered most complications unrelated to the treatment, attributing serious adverse events to cemiplimab in only seven patients (9 %), with pneumonitis in three patients (4 %). Only one death, due to aspiration pneumonia, was attributed to cemiplimab. A final update of results published as a poster presentation,^29^ echoed similar adverse events data, with 192 (99.5 %) of 193 patients experiencing at least one treatment-emergent adverse event. Fatigue was most common in 67 patients (34.7 %), followed by diarrhea in 53 patients (27.5 %) and nausea in 46 patients (23.8 %). Adverse events of grade 3 or 4 were reported in 90 patients (46.6 %), again with hypertension most common in 9 patients (4.7 %), and there were five deaths (2.6 %).

In the KEYNOTE-629 trial^12^ assessing pembrolizumab for advanced cSCC in 159 patients, 110 patients (69.2 %) experienced a treatment-related adverse event, as attributed by study investigators. Grade 3 or 4 adverse events were seen in 17 patients (10.7 %), and 2 patients (1.3 %) died. A trial of neoadjuvant cemiplimab before surgical resection of head and neck cSCC in 79 patients showed similar safety data, with adverse events related to immunotherapy in 57 patients (72 %).^13^ Of these, only seven (9 %) were grade 3 or higher, and there was one death (1 %) attributed to immunotherapy.

This study had several limitations that may have an impact on interpretation of the results. Selection bias is a common consequence of retrospective studies, but the current study used prespecified inclusion and exclusion criteria to minimize this risk. This study did not use randomization, which may have facilitated confounding by case complexity, with higher-stage, more complex cases treated more aggressively, which may have caused regression to the null. Altogether, given the relative lack of exclusion criteria and the varied group of participants, the results of this study will be applicable to most patients with head and neck cSCC who require orbital exenteration.

With the limitations of this study considered, we have shown that for our cohort, older age, residual disease after resection, and lack of postoperative radiotherapy were associated with greater mortality after orbital exenteration for cSCC. Survival rates were good for surgery combined with postoperative radiotherapy. Sole treatment with immunotherapy is yet to achieve comparable mortality results. However, trials are currently limited by inclusion of patients with severe disease who are ineligible for surgery and radiotherapy, which yields a different patient cohort. Adverse events from immunotherapy are significant, but these are likely to be substantially reduced if immunotherapy is included as part of the treatment paradigm (e.g., as neoadjuvant therapy with reduced dosing vs sole treatment). Furthermore, it is difficult to quantify how the adverse effects of orbital exenteration, such as loss of vision, functional debility, and psychosocial harm, compare with the adverse effects of immunotherapy, but these are not trivial. Emerging trial data^13,14^ seem to favor neoadjuvant immunotherapy followed by orbit-preserving wide local excision with or without postoperative radiotherapy.
